# Online Information on Probiotics: Does It Match Scientific Evidence?

**DOI:** 10.3389/fmed.2019.00296

**Published:** 2020-01-15

**Authors:** Marie Neunez, Michel Goldman, Pietro Ghezzi

**Affiliations:** ^1^Institute for Interdisciplinary Innovation in Healthcare, Université Libre de Bruxelles, Brussels, Belgium; ^2^Department of Medicine, Brighton and Sussex Medical School, Brighton, United Kingdom

**Keywords:** probiotics, safety, regulatory, health claims, online, evidence-based medicine, health information

## Abstract

Probiotics are over-the-counter products marketed for enhancing human health. Online information has been key in promoting probiotics worldwide. However, only few rigorous clinical studies have met the stringent criteria required to establish the efficacy and safety of probiotics. The present study was undertaken to assess the information quality of webpages referring to probiotics and to compare the recommendations available online with the information collected from trusted scientific sources. We evaluated 150 webpages returned by Google searching “probiotics” in terms of typology of website, health information quality based on the JAMA score and the HONcode certification, as well as completeness of the information based on the presence of four criteria: (1) links to scientific references supporting health claims, (2) cautionary notes about level of evidence for alleged benefits, (3) safety considerations, and (4) regulatory status. We then enumerated the health claims mentioned online and the corresponding clinical trials and reviews registered in the Cochrane library. Finally, the conclusions of Cochrane reviews were used to assess the level of scientific evidence of the information available through Google search. HON-certified websites were significantly more frequent in the top 10 websites than in the remaining websites. In terms of completeness of information, only 10% of webpages met all four criteria, 40% had a cautionary note on benefits, 35% referred to scientific literature, and only 25% mentioned potential side effects. The results of the content analysis led us to conclude that: (1) the most frequent typologies of webpages returned by Google are commercial and news, (2) commercial websites on average provide the least reliable information, and (3) significant numbers of claimed benefits of probiotics are not supported by scientific evidence. This study highlights important biases in the probiotics information available online, underlining the need to improve the quality and objectivity of information provided to the public.

## Introduction

The World Health Organization defines probiotics as “live microorganisms which, when administered in adequate amounts, confer a health benefit to the host” (1). Although, the association between probiotics and health has already been formulated at the beginning of the twentieth century by Elie Metchnikoff, Nobel Prize Winner in Physiology (2), the development of probiotics as health products is quite recent, in relation with the growing interest in the microbiome (3, 4).

The US probiotics market was estimated to be over 40 billion $ in 2017 whereas the European market is trailing behind, probably due to the stricter regulation for the nutrition and health claims on food supplements [Regulation (EC) No. 1924/2006] (5). Nevertheless, the probiotics market continuously expands with the globalization of online sales. Indeed, together with other over-the-counter medicinal products, probiotics are increasingly popular and widely advertised on the Internet (6). It is therefore important to assess the trustworthiness of the probiotics information that can be found online. For this purpose, we took advantage of an established methodology previously used to analyze health information quality on antioxidants and vaccines (7–9).

Herein, data collected from 150 webpages returned by Google when searching for the term “probiotics” were analyzed for their accuracy and completeness. The Google search engine was used since it is the most used worldwide, with a 75% market share throughout 2017 (10). We then compared the results of this analysis with the information available in the Cochrane library (11), an established source of evidence-based medical information.

## Methods

### Data Collection

The search term “probiotics” was entered on July 23, 2018 in http://google.com using the browser Google chrome (Google LLC, Mountain View, CA, USA) after logging out from any Google account, clearing caches and browsing history to avoid the results to be influenced by previous searches and browsing behavior [the so-called “bubble effect”]. The search was performed from Brussels, Belgium. The first 200 URL returned in the search engine result page (SERP) were transferred to a spreadsheet using the Google extension SEOquake (SEMrush, Trevose, PA, USA). Each URL was then visited and assessed until we reached a total of 150 websites eligible for the study. Exclusion criteria were duplicates, irrelevant websites, websites with paywalls or requiring log-in, video lasting more than 15 min, or dead links.

### Classification of Websites

The 10 URLs that appeared first upon Google search were qualified as “top 10.” Assessment of each URL was performed according to the following parameters:

1. Classification of the website according to typology as follows: commercial (C), governmental (G), news (N), health portal (HP), non-profit organization (NP), professional (P), scientific journals (SJ), and other (O) as previously described (11) (examples of this classification are provided in Supplementary Table 1). This classification was performed independently by two researchers (M. N. and P. G.). Inter-rater variability was assessed using GraphPad. The observed agreements were 133 of 150, reaching a very good strength of agreement [89% of the observations, resulting in a weighted kappa = 0.841, 95% confidence interval (0.772, 0.910)]. The final typology resulted from a consensus between the two researchers.
2. The Journal of the American Medical Association (JAMA) score. This highlights the presence of four elements: authors' name, date of publication or update, indication of the website's owner, and references to sources (12). Each criterion scores 1 unit, so that the JAMA score ranges from 0 to 4. The JAMA score classification was performed by one author (MN) and double checked by a second author (PG), and disagreements discussed and resolved.
3. The presence or absence of the HONcode seal on the page. The HONcode certification is provided by an independent organization (Health-on-the-net, Lausanne, Switzerland) and addresses the reliability and the credibility of the information found on the website based on eight criteria: authoritativeness (qualifications of the authors), complementarity (supporting but not replacing doctor-patient relationship), privacy, attribution (citing the sources), justifiability (baking claims relating to benefits), transparency (contact information and identity of editor and webmaster), financial disclosure, and a clear distinction of adversisements from editorial content (13). Of note, the JAMA score and the HONcode are trustworthiness indicators that do not rate the content of the information provided by the website (13). The presence of the HONcode seal was assessed by one author (MN) and cross-validated by a second author (PG).
4. The diseases or biological processes [e.g., skin health, mental health, cardiovascular diseases (CVD), gastrointestinal health, cancer, uro-genital health, immune system support, and respiratory health] mentioned in the context of potential benefits of probiotics. This list was compiled based on the indications mentioned in the webpages
5. The species of the microorganisms present in the probiotics mentioned.
6. The completeness of the scientific information found on each webpage. For this, we looked for mentions regarding (i) regulatory framework; (ii) relevant scientific documentation; (iii) caution about potential benefits; and (iv) potential side effects of probiotics. Each item was given a score of “1” so that the completeness score ranged from 0 to 4. Examples for this classification are provided in Supplementary Table 2.

A requirement for scoring webpages for any of the items above was that the information had to be available within three clicks. The rationale behind is that information quality is studied from the perspective of the lay public who will unlikely go beyond three clicks to search for information (14).

It should be noted that a webpage could mention more than one disease or biological process, and more than one microorganism. These were considered and reviewed separately.

### Comparison of Online Claims With Evidence-Based Information in the Cochrane Library

We used the information in the Cochrane library (10) as a proxy for the strength of the scientific evidence available on the health benefits of probiotics in specific indications. As mentioned in Cochrane website, Cochrane review attempt to identify, appraise and synthesize all the empirical evidence that meets pre-specified eligibility criteria to answer a specific research question (15). We recorded the numbers of Cochrane reviews as well as the numbers of clinical trials present in the Cochrane library for each of the indications mentioned in the SERP. Conclusions on the evidence-based benefits of probiotics for the quoted indications were drawn from the abstracts of the reviews. The analysis was completed by the end of February 2019.

### Statistical Analysis

Data referring to categorical variables (scores) are expressed as median and interquartile range (IQR). To compare categorical variables (JAMA score, completeness score in different typologies of websites a non-parametric two-tailed Kruskal-Wallis multiple comparison test, followed by the Dunn's post test was used. To compare the completeness score in two groups (e.g., commercial vs. non-commercial websites), a Mann-Whitney test was used.

Comparison of the frequency of website typologies in the top 10 webpages returned by Google vs. the remaining 140 webpages was dove using a two-tailed Fisher's exact test. This was also used when comparing the frequency of HONcode-certified webpages in the top 10 results vs. the remaining 140 websites. The statistical analysis was pre-defined and followed exactly the same design used in our previous studies (7–9).

## Results

### Distribution of Websites by Typology

**Figure 1:**
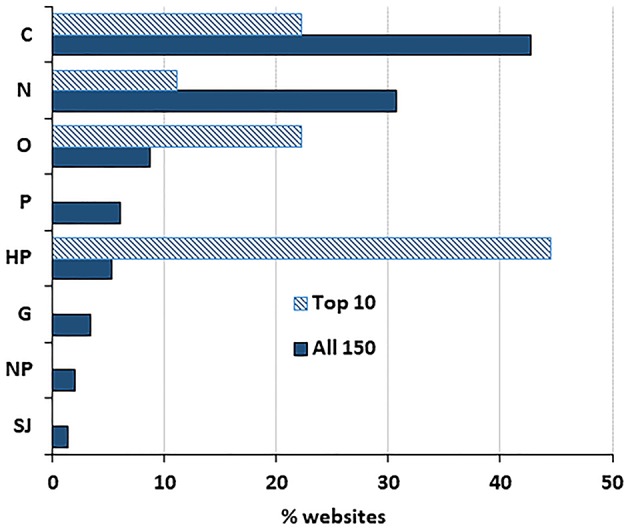
Distribution of websites by typology. Data show the percentage of websites in the top 10 results (*n* = 9, as one of the websites ranked in the top 10 by Google was a duplicate) and the total number of websites in the search (*n* = 150).

Of the 150 websites analyzed, the most frequent typology was “commercial” websites (43%) followed by “news” (31%); all other typologies present accounted for <10% (Figure 1). A different pattern was observed for the top 10 webpages returned by Google, where the most frequent typology was “health portal” (44%), followed by “commercial” (22%). This over-representation of “health portals” was statistically significant (*P* < 0.0005 by a Fisher's test).

### Analysis of Trustworthiness Criteria: Jama Score

The overall trustworthiness of each webpage was assessed by calculating the JAMA score as defined in the Methods section. The median JAMA score was 3, IQR [2.5, 4], and was not significantly different in the top 10 websites.

**Figure 2:**
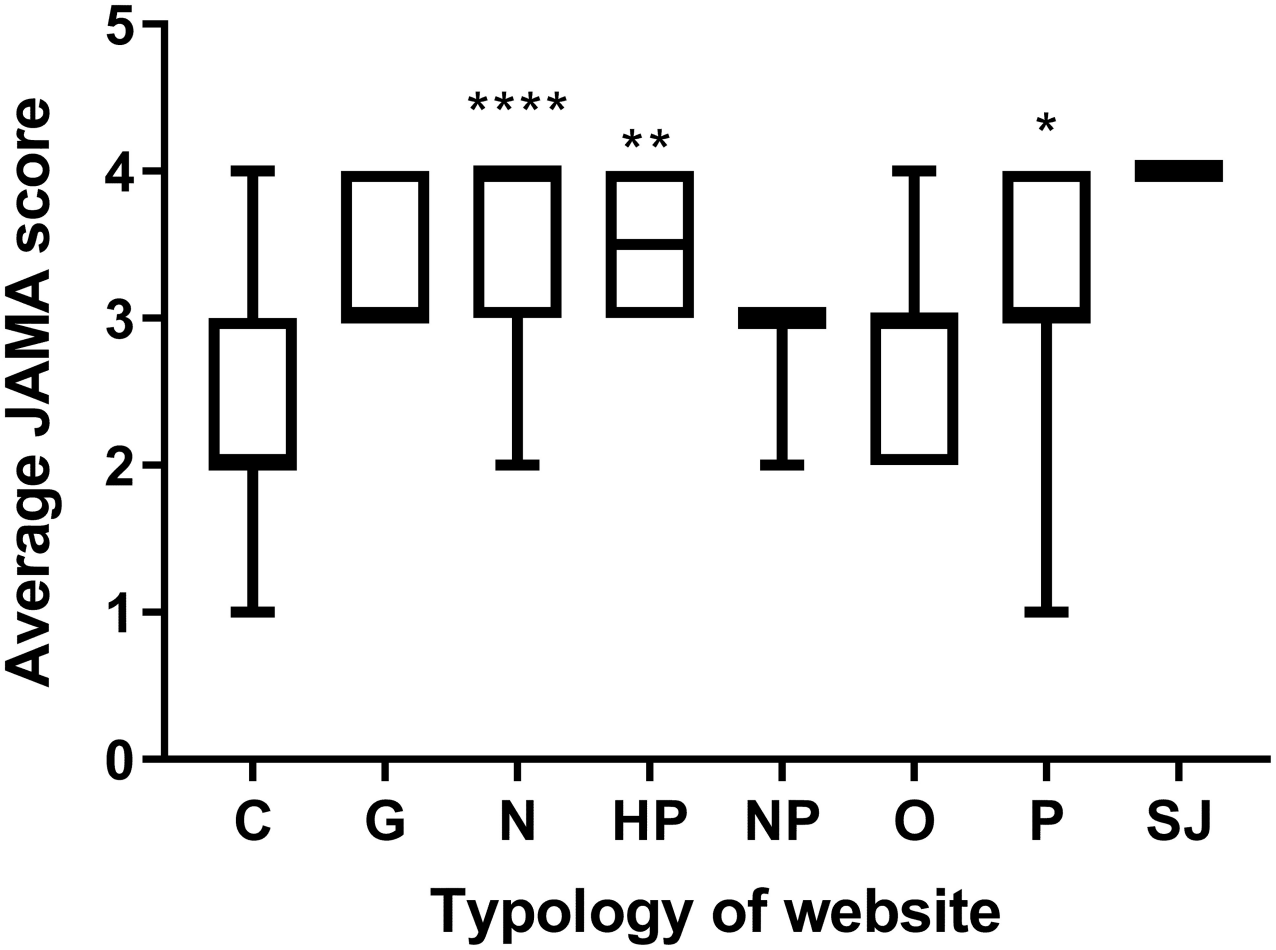
JAMA score by typology of website. Data are reported as median and interquantile range. Significantly different commercial websites (*P < 0.05, **P < 0.005, ****P < 0.0001; two-tailed Kruskal-Wallis multiple comparison test, followed by the Dunn's post test) Number of websites: C, 64; G, 5; N, 46; HP, 8; NP, 3; O, 13; P, 9; SJ, 2.

Figure 2 displays the median JAMA score of websites according to the different typologies. Commercial websites had the lowest JAMA score of all typologies, with a significantly lower median than professional, health portal, and news websites.

### Analysis of Trustworthiness Criteria: HONcode Certification

In total, only 13 websites displayed the HONcode certification. The frequency of websites certified by HONcode was significantly higher in the top 10 websites (4/9, 44%) than in the remaining websites (9/141, 6%; *P* < 0.005 by a two-tailed Fisher's test).

### Completeness of Information

**Figure 3:**
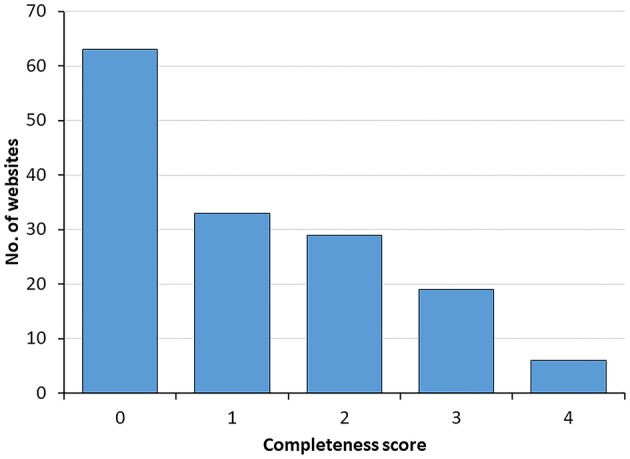
Distribution of websites according to the completeness of scientific information score. Data illustrate the number of websites ranking from 0 to 4 in the completeness score.

Completeness of the scientific information available on websites was evaluated based on the following four criteria: (1) links to scientific references supporting health claims, (2) cautionary notes about level of evidence for alleged benefits, (3) safety considerations, and (4) regulatory status. As shown in Figure 3, most websites provide poor information, with over 60% scoring zero and <10% scoring positively for the four criteria. When the four criteria were analyzed separately, 40% of webpages had a cautionary note about probiotics health benefits suggesting that additional research need to be done, 35% were referencing to scientific literature when mentioning defined probiotics indications, 25% mentioned potential side effects, whereas only 15% mentioned regulatory provisions.

Of note, the completeness score was significantly higher in the top 10 websites (median 2, IQR [1, 3.5]) than in the remaining websites (median 1, IQR [0, 2]) (*P* < 0.005 using Mann-Whitney's test).

**Figure 4:**
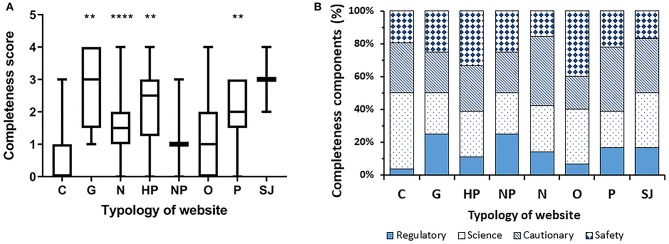
Completeness score by typology of website. **(A)** Data are reported as median and interquantile range (**P < 0.005, ****P < 0.0001; significantly different from commercial websites by a two-tailed Kruskal-Wallis test, followed by the Dunn's post test). **(B)** Breakdown of the four components per typology.

Figure 4 depicts level of completeness by website typology. Multiple comparison showed that commercial websites had the lowest completeness score. The median score for commercial websites was significantly lower than that of non-commercial websites (median 0, IQR [0, 3] vs. median 2, IQR [0, 4]; *P* < 0.0001 by Mann-Whitney's test). The highest completeness score was observed in governmental websites and scientific journals' websites.

The information about the exact content of the probiotic products was highly variable. Of note, 29 out of the 150 webpages (19%) did not provide any information on the bacterial strains composing probiotics.

### Comparing Information Online and Evidence-Based Information From the Cochrane Organization

**Figure 5:**
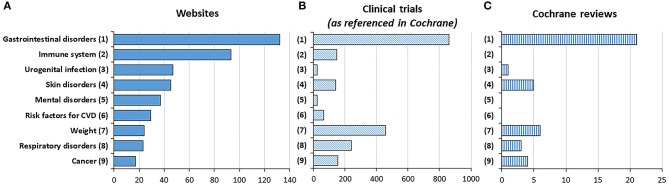
Number of websites, trials, and meta-analyses in the Cochrane database by indications. Data indicates the number of occurrences of an indication in websites **(A)**, in clinical trials **(B)**, and in reviews **(C)**.

To get further insight in the accuracy and completeness of the information contained in the 150 analyzed webpages, we first extracted and ranked the clinical settings for which probiotics were claimed to be beneficial (Figure 5A). This was done referring to the nine therapeutic areas described in the methods. Of the 150 websites, nine did not make any claim about their usefulness in a disease while only one mentioned all nine types for indication analyzed. The median number of claims mentioned was 3, IQR [1.75, 4]. We then enumerated the number of clinical trials registered in the Cochrane Central Register of Controlled Trials (Figure 5B), as well as the number of reviews performed by Cochrane Review Groups (Figure 5C). Gastro-intestinal disorders are the most often referred online claims (132 websites, 88%) and also the subject of the highest number of clinical trials and Cochrane reviews. In contrast, immune enhancement which is the second most referred online claim (93 websites, 62%) has been barely investigated in clinical trials and has not been reviewed at all by Cochrane Review Group. A similar situation is observed for mental disorders and risk factors for cardiovascular diseases, with claims which are not supported by any Cochrane review.

For each claim (which refers to a therapeutic indication), we extracted the level of scientific evidence based on the terminology used by the Cochrane Review Group (Table 1). Among gastrointestinal disorders, Cochrane reviews support online claims regarding infectious diarrhea, including Clostridium difficile-associated colitis. However, there is still uncertainty about which probiotics should be used for which groups of people, and also to assess the cost effectiveness of this treatment. Cochrane reviews also support the use of probiotics in the prevention of necrotizing enterocolitis in preterm infants, but with insufficient data regarding the benefits and the potential adverse effects in the most at risk infants. In contrast, no evidence was found in Cochrane reviews regarding the benefits of currently used probiotics in cancer, obesity and respiratory disorders. For the other indications, the level of evidence is low or moderate.

**Table 1:** Level of scientific evidence for online health claims for probiotics.

**Clinical setting**		Evidence^*^	N Google pages^**^
GI disorders	Infectious diarrhea (including Clostridium difficile colitis)	✓	24
	Necrotizing enterocolitis	✓	18
	Irritable bowel syndrome	≈	41
	Antibiotic-associated diarrhea	≈	45
	Ulcerative colitis	~	16
	Pouchitis	~	6
	Crohn's disease	~	8
	Food intolerance	~	23
Urogenital disorders	Urinary	~	21
	Vaginal	~	35
Skin disorders	Eczema	~	22
Weight disorders		×	24
Respiratory disorders		×	23
Cancer	Colorectal	×	9
	Bladder	×	4
	Liver	×	2
	Lung	×	1
	Stomach	×	1
	Breast	×	1
	Cervical	×	1

* *Derived from conclusions of Cochrane reviews. green (✓), established evidence; yellow (≈), moderate evidence; orange (~), low evidence; red (×), no evidence*.

** *Number of health claims per clinical setting within the 150 websites analyzed*.

**Figure 6:**
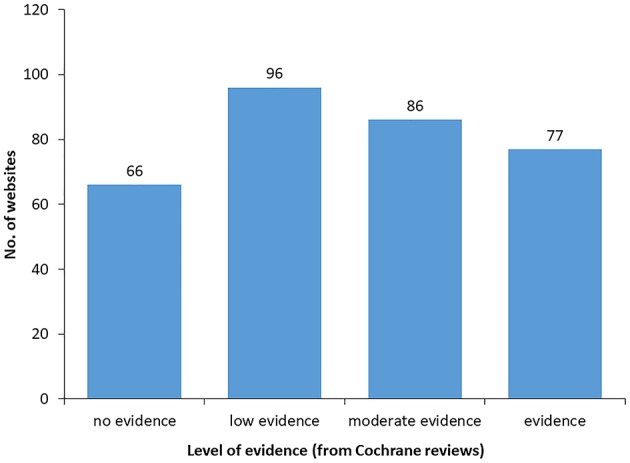
Number of online claims with different levels of scientific evidence according to Cochrane reviews. Values do not add up to 150 as webpages can mention more than one probiotics' benefit.

Finally, for each of the four levels of evidence in Cochrane reviews we enumerated the number of webpages referring to probiotics health claims. As shown in Figure 6, only 77 out of the 325 online claims (23%) are supported by substantiated scientific evidence according to Cochrane reviews. On the other hand, 66 out of 325 online claims (20%) are not supported by any scientific evidence in the current state of knowledge.

## Discussion

Health literacy is increasingly important to ensure that citizens take the best advantage of marketed health products. In the current era where distrust in medical experts and health authorities is widespread, individual consumption of over-the-counter health products is largely guided by information collected on the Internet. Since probiotics escape scrutinization by regulatory authorities, it is of utmost importance to get insight into the level of trustworthiness provided by online information on their benefits and risks.

First, we observed that a high proportion (43%) of the websites returned by Google search on probiotics are of commercial nature, although these had a lower ranking as there were only 22% commercial websites in the top 10 page returned by Google. Commercial websites scored the lowest both in terms of JAMA score and HONcode certification. Although “health portal” and “news” websites seem more trustworthy according to these criteria, the information they provide might still be biased by the interest of their private sponsors. One might assume that governmental websites supported by public sources might be the most reliable source of information but unfortunately, they are few and none is returned among the top 10 upon Google search.

We then investigated the completeness score of the webpages by a methodology based on four main criteria, as in a previous study on a different topic (16). Strikingly, over 60% of webpages scored 0 whereas <10% scored 4, with commercial websites again ranking in the lowest range (Figure 3). The top 10 websites showed a significantly higher completeness score indicating that this aspect of information quality is reflected in the Google algorithm used for the ordering of websites. In terms of consumer protection, information on the potential risks associated with the use of probiotics is especially important. Unfortunately, only 25% of webpages (and only 8% of the commercial ones) include safety considerations and refer to possible side effects. Moreover, the assessment by regulatory authorities is mentioned in only 15% of all webpages, and 2% of the commercial ones. As a matter of fact, claims on the benefits and risks of probiotics in human diseases have not been approved neither by the US Food and Drug Administration (FDA) nor the European Food Safety Authority (EFSA) that are responsible for probiotics regulation in US and Europe, respectively. Although the overall safety profile of probiotics seem favorable, there are isolated reports of fungemia and bacteremia related to probiotics administration in immunocompromised individuals including neonates with very low birth weights (17, 18). The latter cases are especially important to consider since prevention of necrotizing enterocolitis is one of the few clinical settings in which the efficacy of probiotics is best established according to Cochrane reviews.

We acknowledge that the current study has limitations which might influence the interpretation of our findings. Obviously, the websites returned by Google search depend on the date of the search and the search terms used. Furthermore, Cochrane reviews might fall short in defining the level of scientific evidence for clinical benefit. They are often based on meta-analyses of trials that are heterogeneous in terms of clinical indications, design, as well as composition and formulation of probiotics compounds. These shortcomings affect both studies published in the scientific literature and the online information. As a consequence, both health professionals and lay people are exposed to incomplete information regarding probiotics.

We conclude that the high level of uncertainty for most health claims found online hinders the rational use of probiotics, leaving the field open to unsubstantiated allegations and misuse. With the growing interest in therapeutic interventions targeting the microbiome, there is a clear need for a new regulatory framework and new policies regarding communication on the benefits and risks of probiotics.

## Data Availability Statement

The raw data supporting the conclusions of this article will be made available by the authors, without undue reservation, to any qualified researcher.

## Author Contributions

MN and PG analyzed jointly the typology of websites. MG and PG contributed to the manuscript draft prepared by MN.

### Conflict of Interest

MG serves as scientific advisor for Vésale Pharma S.A. The remaining authors declare that the research was conducted in the absence of any commercial or financial relationships that could be construed as a potential conflict of interest.
