# Supplementary material for: Online Information on Probiotics: Does It Match Scientific Evidence?
Source: Front Med (Lausanne). 2020 Jan 15;6:296. doi: 10.3389/fmed.2019.00296 (PMC6974687; doi:10.3389/fmed.2019.00296)
Supplement: Supplementary file 1 [file Data_Sheet_1.PDF]

SUPPLEMENTARY FILES

Typology	Examples
Commercial (C)	https://draxe.com<br><br>https://www.humnutrition.com
Governmental (G)	https://nccih.nih.gov<br><br>https://www.nasa.gov
News (N)	https://www.womenshealthmag.com<br><br>http://time.com<br><br>https://www.theguardian.com
Health portal (HP)	https://www.webmd.com<br><br>https://www.medicinenet.com
Non-profit organization (NP)	https://nutritionfacts.org
Professionals (P)	https://my.clevelandclinic.org<br><br>https://www.health.harvard.edu
Scientific journals (SJ)	https://www.sciencedirect.com<br><br>https://www.scientificamerican.com
Other (O)	https://en.wikipedia.org<br><br>https://blog.paleohacks.com

Supplementary Table 1. Examples of websites typologies

Criteria	Examples	Websites
Cautionary note on probiotics health benefits	- Kombucha is a fermented tea drink. It is claimed to have a wide range of health benefits, but more research is needed. (https://www.healthline.com/nutrition/11-super-healthy-probiotic-foods)<br>- However, benefits have not been conclusively demonstrated, and not all probiotics have the same effects. (https://nccih.nih.gov/health/probiotics/introduction.htm)	
Relevant scientific documentation	- https://www.healthline.com/nutrition/11-super-healthy-probiotic-foods<br>- https://nccih.nih.gov/health/probiotics/introduction.htm	
Safety information on probiotics	- In general, probiotic foods and supplements are thought to be safe for most people, though some people with immune system problems or other serious health conditions shouldn't take them. (https://www.webmd.com/digestive-disorders/what-are-probiotics)<br>- What the Science Says About the Safety and Side Effects of Probiotics (https://nccih.nih.gov/health/probiotics/introduction.htm)	
Regulation on probiotics	- The FDA regulates probiotics like foods, not like medications. Unlike drug companies, makers of probiotic supplements don't have to show their products are safe or that they work. (https://www.webmd.com/digestive-disorders/what-are-probiotics)	

Supplementary Table 2. Examples that satisfy each criterion of the score of completeness
